# The complete mitochondrial genome of the edible mushroom *Pleurotus giganteus* (Agaricales, Pleurotus) and insights into its phylogeny

**DOI:** 10.1080/23802359.2022.2096418

**Published:** 2022-07-18

**Authors:** Zengliang Liu, Shengjin Wu, Xuefeng Chen, Wenlong Zhang, Shuangyun Zhou, Xiaoguo Wang

**Affiliations:** a Microbiology Research Institute, Guangxi Academy of Agricultural Sciences, Nanning, China; b Horticulture Research Institute, Guangxi Academy of Agricultural Sciences, Nanning, China

**Keywords:** *Pleurotus giganteus*, mitochondrial genome, phylogenetic relationship

## Abstract

*Pleurotus giganteus* (Berk.) Karunarathna & K.D. Hyde 2011 is one of the largest edible mushrooms integrating medicinal value and edible value. The complete mitochondrial genome of the edible fungus *P. giganteus* was published in this paper. It was determined using Pacbio and Illumina sequencing. The circular molecule is 102,950 bp in length, consisting of 30 protein-coding genes (PCGs), two ribosomal RNA (rRNA) genes, and 24 transfer RNA (tRNA) genes. The base composition of the whole mitogenome is A (37.3%), T (37.7%), G (12.2%), and C (12.8%). The phylogenetic tree shows *P. giganteus* was the basal taxon in *Pleurotus* and closely related to *Pleurotus citrinopileatus* Singer 1990.

*Pleurotus giganteus* (Berk.) Karunarathna & K.D. Hyde 2011, previously reported as *Lentinus giganteus* or *Panus giganteus*, has been used as a culinary mushroom and is increasing in popularity for its medicinal properties and commercial prospects (Baskaran et al. 2017). *P. giganteus* has been recorded in Sri Lanka (Klomklung et al. 2012), Thailand (Klomklung et al. 2012), Laos (Phonemany et al. 2021), China (Bi et al. 1993; Phan et al. 2012), and Oceania (Bi et al. 1993). *P. giganteus* has high contents of magnesium, potassium, amino acids, iron, and calcium which may benefit human health (Phan et al. 2014, 2019). Furthermore, *P. giganteus* has been reported to be containing stimulation of neurite outgrowth (Phan et al. 2012), possessing liver protection properties (Wong et al. 2012), was a healthy dietary supplement for brain and cognitive health (Phan et al. 2013), and inhibition of growth of *Candida* species (Phan et al. 2013).

Mitochondria are presumed to be derived from bacteria through endosymbiosis (Muñoz-Gómez et al. 2017). The mitochondrial genome contributes to systemic evolution, population genetics, and taxonomy (Carpi et al. 2016; Ramos et al. 2018). However, no complete mitogenome is available to date for *P. giganteus*. Here, we report the complete mitogenome of *P. giganteus* using next-generation sequencing, which might provide new insights into genetic structure and differentiation of this species (Figure 1).

**Figure 1.**
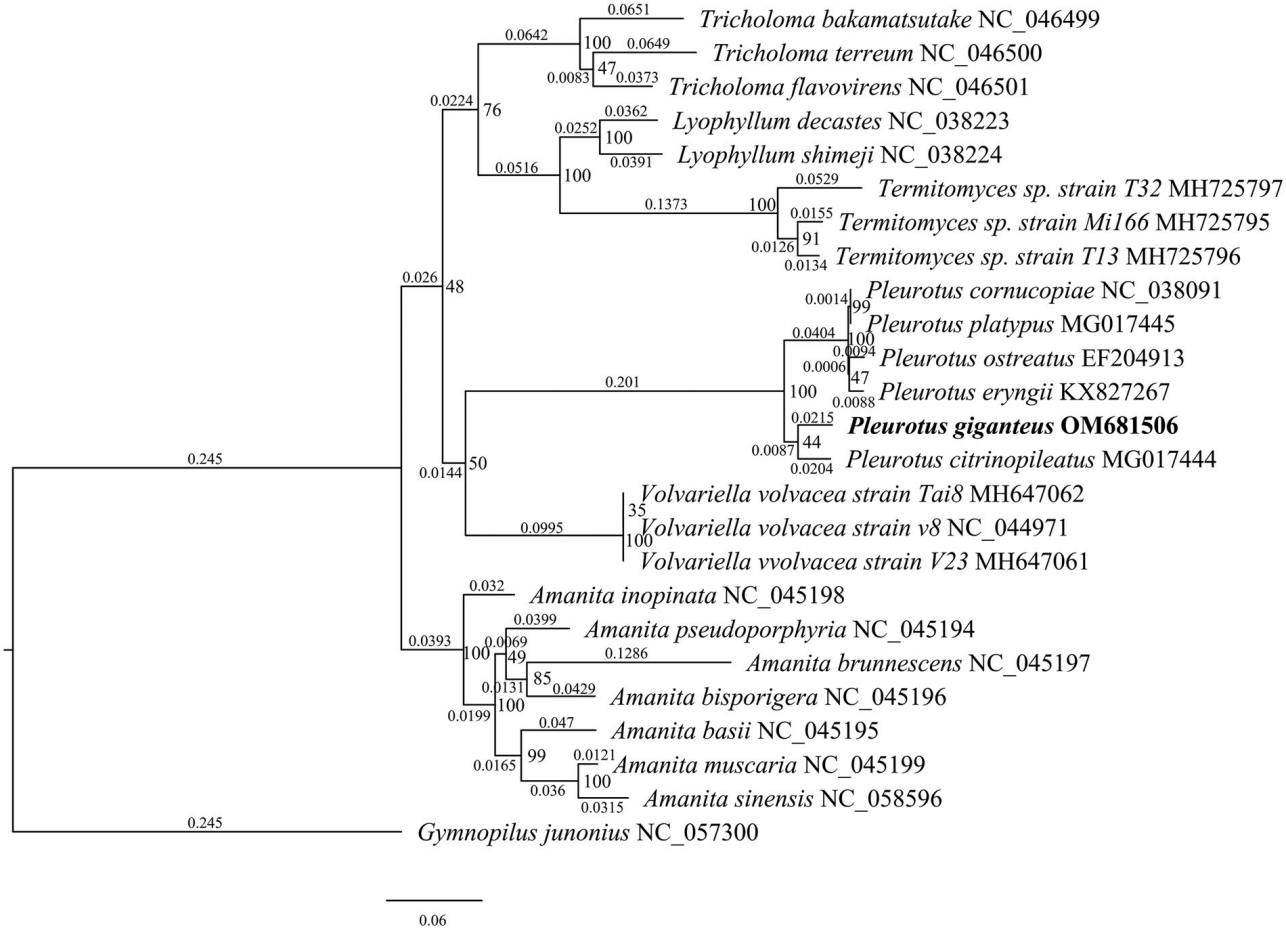
The ML phylogenetic tree of 24 species conducted based on the amino acid dataset of eight mitochondrial protein-coding genes, including cox1, rps3, nad6, cob, atp9, cox3, nad4L, and nad5.

The specimen of this study was mushroom and no ethical issues are involved. The study has been granted an exemption from requiring ethical approval by the Committee on the Ethics of Microbiology Research Institute, Guangxi Academy of Agricultural Sciences, Nanning, China. The specimen used in this study was collected from artificial cultivation in Nanning, Guangxi Province, China (108.24E, 22.84N) with the permission granted by Guangxi Academy of Agricultural Sciences, and it was stored in Guangxi Academy of Agricultural Sciences Herbarium (voucher specimen: MG-GX2020024, Zengliang Liu, zengguang201010@163.com). The specimen was identified as *P. giganteus* by morphology, internal transcribed spacer (ITS) sequence and small subunit ribosomal RNA (rRNA) (rns) sequence. The living culture was deposited at Institute of Microbiology, Guangxi Academy of Agricultural Sciences (no. WZDBX006, Zengliang Liu, zengguang201010@163.com). The mitochondrial genome of *P. giganteus* was obtained by Illumina sequencing technology (Novaseq 6000, San Diego, CA) and assembled in SPAdes v. 3.11.0 (Bankevich et al. 2012). The original annotation of mitochondrial genome was obtained from a sequencing laboratory: Huitong Biotechnology (Shenzhen, China). MITOS (http://mitos.bioinf.uni-leipzig.de/index.py) was used for mitochondrial genome annotation (Bernt et al. 2013).

The complete mitochondrial genome of this mushroom is a circular DNA of 102,950 bp in length with a GC content of 25.0% (GenBank: OM681506). The base composition of the *P. giganteus* mitochondrial genome is as follows: A (37.3%), T (37.7%), G (12.2%), and C (12.8%). The mitochondrial genome of *P. giganteus* contained 56 genes including 30 protein-coding genes (PCGs), two rRNA genes (rnl and rns), and 24 transfer RNA (tRNA) genes. The 30 PCGs encoded 14 conserved mitochondrial proteins (cox1-3, cob, nad1-6, nad4L, atp6, atp8, and atp9) and a ribosomal protein S3. There were 15 introns distributed in two PCGs, i.e. cob (three introns) and cox1 (12 introns).

We used OrthoFinder v2.3.14 (Emms and Kelly 2019) to select eight homologous single-copy PCGs in 24 species with *P. giganteus* from NCBI database, then we aligned them with *P. giganteus* by using muscle v3.8.1551 (Edgar 2004). The best substitution model was tested based on the Bayesian information criterion (BIC) by prottest v3.4 (Darriba et al. 2011). The best-fitting model in the analysis was CpREV + I+G + F. Maximum-likelihood analysis was performed in RAxML v.8.2.12 (Stamatakis 2014) with 1000 rapid bootstrap analyses, followed by a search for the best-scoring tree in one single run. *Gymnopilus junonius* was used as outgroup. The analysis confirmed that *P. giganteus* was a member of *Pleurotus* and closely related to *Pleurotus citrinopileatus*. The complete mitochondrial genome sequence of *P. giganteus* will be helpful for further studies on population genetics, taxonomy, or resource protection.

## Author contributions

Study conception and design: Zengliang Liu and Shengjin Wu; data collection: Xuefeng Chen; analysis and interpretation of results: Wenlong Zhang and Shuangyun Zhou; draft manuscript preparation: Zengliang Liu and Xiaoguo Wang; revising it critically for intellectual content: Xiaoguo Wang and Shengjin Wu. All authors reviewed the results and approved the final version of the manuscript. All authors agree to be accountable for all aspects of the work.

## Data Availability

The genome sequence data that support the findings of this study are openly available in GenBank of NCBI (https://www.ncbi.nlm.nih.gov/) under the accession number OM681506. The associated BioProject, SRA, and Bio-Sample numbers are PRJNA818472, SRR18426954, and SAMN26863501, respectively.
